# The Prediction of Consumer Behavior from Social Media Activities

**DOI:** 10.3390/bs12080284

**Published:** 2022-08-12

**Authors:** Nada Ali Hakami, Hanan Ahmed Hosni Mahmoud

**Affiliations:** 1 Computer Science Department, College of Computer Science and Information Technology, Jazan University, Jazan 82822-6694, Saudi Arabia; 2 Department of Computer Sciences, College of Computer and Information Sciences, Princess Nourah bint Abdulrahman University, P.O. Box 84428, Riyadh 11671, Saudi Arabia

**Keywords:** consumer behavior, social media activities, deep learning

## Abstract

Consumer behavior variants are evolving by utilizing advanced packing models. These models can make consumer behavior detection considerably problematic. New techniques that are superior to customary models to be utilized to efficiently observe consumer behaviors. Machine learning models are no longer efficient in identifying complex consumer behavior variants. Deep learning models can be a capable solution for detecting all consumer behavior variants. In this paper, we are proposing a new deep learning model to classify consumer behavior variants using an ensemble architecture. The new model incorporates two pretrained learning algorithms in an optimized fashion. This model has four main phases, namely, data gathering, deep neural modeling, model training, and deep learning model evaluation. The ensemble model is tested on Facemg BIG-D15 and TwitD databases. The experiment results depict that the ensemble model can efficiently classify consumer behavior with high precision that outperforms recent models in the literature. The ensemble model achieved 98.78% accuracy on the Facemg database, which is higher than most machine learning consumer behavior detection models by more than 8%.

## 1. Introduction

In recent years, it is feasible to perform everyday activities using the Internet including social media interaction. All of these activities include consumers submitting reviews online [1,2,3]. Consumers often use the Internet to launch consumer-related communication. Consumer behavior is any message or social media-based communication that performs reviewing language. Consumer behavior can be classified into various types. Consumer behavior variants can include consumer reviews. New consumer behavior variants utilize various models such as encryption and packing to remain visible to consumer reviews system [3].

We have to detect consumer behavior as soon as it spreads into the social media platform. Consumer behavior prediction is the procedure of investigating review messaging in social media interactions and predicting if it is consumer/non-consumer behavior. Consumer behavior can be classified as immediate or future. Identifying consumer behavior requires three steps:

(1) Consumer behavior messages and social media interaction are analyzed with proper tools.
(2) Dynamic features such as timing are extracted the interaction data.
(3) Parameters are assembled in specified sets and are used to differentiate consumer from non-consumer behavior.

To enhance the detection rate, different techniques such as data science, cloud computing, deep learning, and computerized learning models are utilized. Various consumer behavior prediction techniques utilize these technologies. These models are signature checking, behavioral analysis and stochastic learning models [2,4]. Signature-checking models are effective for identifying similar variants of consumer behavior. However, they fail to detect formerly unnoticed consumer behavior. Although stochastic models can detect unknown consumer behavior, they cannot detect more complex consumer behavior clarifications.

A deep learning model can be utilized as a standard to eradicate the shortcomings of the current consumer behavior classification models. Deep learning is utilized extensively in different paradigms such as representation processing, human emotion recognition [5], and action recognition [6,7,8]. Nevertheless, it has not been utilized adequately in the retail research, particularly consumer behavior detection. Deep learning is an artificial intelligence model operating on an artificial neural mechanism. Deep learning employs supervision. To enhance the precision, various models have been utilized such as deep belief techniques. Deep learning models have many advantages over customary models: for instance, deep learning models can mine significant data from the input to lessen the training requirements. Deep learning can also use representations resourcefully as well processing big databases while reducing time and enhancing precision.

Our research presents a new ensemble learning model for consumer Internet behavior classification. In the ensemble model, consumer behavior data are collected from BIG-D15, Facemg, and TwitD databases. Consumer behavior representations are transformed into grayscale and then used as input to the training module. After the data procurement section is complete, the ensemble model extracts high-level consumer behavior features from the consumer behavior representations by utilizing the convolution function of our ensemble model. The model then undergoes supervised training. Several deep models are united to build the ensemble learning model using a number of hidden layer activation functions. The experiments performed prove that the ensemble model efficiently mines unique properties for consumer behavior groups. The performance also presented that the ensemble model predicts various consumer behavior classes with the utmost precision with better performance than recent models.

The main contributions of our research are as follows:

1. A new ensemble neural model for consumer Internet behavior classification is defined.
2. The ensemble model utilizes a new merging technique that has two transfer learning CNNs.
3. Unique parameters are mined from the consumer behavior data for the specified classes.
4. The ensemble model lessens the parameter dimensionality considerably.
5. The ensemble model evaluates consumer behavior databases.
6. The performance rates outperform similar models.

The rest of the article is structured as follows: In Section 2, we clarify the consumer behavior investigation, parameter selection, and classification and survey the current consumer behavior prediction models. The ensemble model is depicted in Section 3. Section 4 presents and discusses the experiment results. Section 5 lists the limitations of the model and future work directions. Conclusions are given in Section 5.

## 2. Related Work

Consumer behavior detection is a long procedure with many phases. Many techniques are utilized in this procedure. The detection of consumer behavior requires analyzing the existing methods. Results are recorded, and parameters are then computed. In the next stage, intelligent methods are utilized to select only the significant parameters [9,10,11]. Selected features are used in the training phase of the neural model to distinguish consumer behaviors. Consumer behavior analysis and classification processes are depicted and summarized. To comprehend the reason for a consumer’s behavior, more prediction is employed by identifying the groups of consumer behaviors [10]. In order to comprehend the consumer behavior mining methods that are applied, this summary is structured as follows: consumer behavior detection platforms, consumer behavior analysis, consumer behavior feature extraction, and classification.

### 2.1. Over the Internet Consumer Behavior Detection Platforms

Consumer behavior detection models can be performed on computer cloud platforms, mobile, and IoT devices. Smart platforms developed widespread paradigms. Then, these platforms started to become available. In current studies, people widely utilize mobile applications, and IoT platforms are expanding rapidly. Therefore, consumers’ interests have moved to Internet computing paradigms [11,12,13]. Cloud computing also aids consumer behavior by simple access to different databases [13]. Recent papers on consumer behavior classification models are concerned with IoT and Internet communication. Most published research paved the way to deep learning techniques.

### 2.2. Consumer Behavior Analysis Model

Consumer behavior samples must be investigated to discover consumer behaviors [14]. Consumer behavior investigation is a significant procedure for online retail. Consumer behavior detection is performed to answer questions such as the nature of the consumer behavior structure, the plan and place where the consumer activity will take place, and the spread network for identifying the review scores. Consumer behaviors are split into two classes, local and global. Consumer behavior undergoes static and dynamic analysis [15].

In static analysis, file texts and representation data shared over the Internet are examined by analyzing data without details [14]. To mine these data, numerous tools can be utilized such as PEiD and MD5deep [15]. Static analysis is the initial stage of consumer behavior analysis; to achieve better understanding, advanced static investigation is recommended. In advanced analysis, the representations and social media interactions are inspected in detail [16,17]. For this reason, complicated representation splitters are extensively used. In the analysis phase, social media interactions and shared representations are inspected in depth to find out features of consumer behavior. Certain consumer behavior maps can be drawn as a result of reversing social interaction networking. However, due to the huge amount of data on the Internet, performing such analysis needs more time [14,18].

In dynamic analysis, reverse interaction maps are executed, and the behaviors of the involved consumers behavior. The consumer behavior is monitored with process explorer [17]. In advanced analysis, tools such as WinOlly are utilized. Such tools allow consumer behavior and Internet interaction to process for both reading and sharing [14].

### 2.3. Consumer Behavior Parameter Setting

When individual consumer actions are studied, the interaction timeline is recorded. Records will be analyzed to mine consumer behavior parameters. Learning models use previous knowledge from big data. At this phase, assured patterns in the data and unknown values are extracted. In recent research, data mining methods such as n-gram, bags and the net model are utilized when identifying consumer behavior parameters [9,10]. There are parameter selection systems that can be presented for consumer behavior prediction [9,10,11,12].

#### 2.3.1. Feature Extraction Methods

N-gram models is utilized in many classification models including consumer behavior classification through sentence analysis. N-gram models can use feature mining to extract learning parameters. Once parameters are selected from the consumer behaviors, it can utilize temporal text analysis [13]. For example, if a sample sentence is S = {1,2,3,4}, 2-gram and 3-gram will be {⟨1,2⟩, ⟨2,3⟩} and {⟨1,2,3⟩, ⟨2,3,4⟩}, respectively. Bag feature extraction models are comparable with n-gram models with high occurrences and are of higher significance than the word location [14]. Although this technique is an operative grouping method, the high growth in the count of the parameters decreases its efficiency. Other versions of this model group consumer behavior features intelligently [14,15,16].

#### 2.3.2. Graph Maps for Feature Extraction

Internet interactions gathered while extracting features include strings, friend networks, messages, and interactions and are represented by diagrams [17,18,19], where the map representation is M (N, E) and N is a vertex representing people in a subset of the social media interactions and E are edges representing interaction among the nodes. The problem is that the graph size can grow exponentially over time, and therefore, we use map subsets to approximate the whole map representation s [19]. However, extracting the map subsets is a hard problem in research, and therefore stochastic or greedy algorithms are employed.

#### 2.3.3. Visual Techniques

Visual parameter mining techniques have several algorithms to mine the parameters of consumer behavior. In one algorithm, consumer behavior text binaries are used as a representation. In this algorithm, a consumer behavior binary is represented and saved as a one-byte vector [19,20,21]. In another algorithm, consumer behavior analysis is performed using tools such as IDA Pro [22]. Then, interaction maps are collected and visualized as representations. In the representation process, models such as graph maps are utilized. In recent research, facts are established that consumer behavior groups and have comparable visual parameters [23,24,25,26].

### 2.4. Consumer Behavior Deep Learning Classification Models

Consumer behavior features are extracted by utilizing deep learning or experimental models to predict the consumer behavior. We divide review score classification models to different models comprising signature, machine and deep learning models [27].

#### 2.4.1. Signature Classification Model

A bit series that represents the interaction map consists of exclusive bits for structures and are utilized in consumer behavior classification [28]. In the computation, the fixed parameters are mined from the interaction data. The computational process produces outputs by utilizing interaction data and stores them in a database. When the reviewing consumer behavior is marked as actual, the signature is computed and compared with the predetermined signatures as actual. This model is efficient in detecting consumer behavior, but it cannot identify unknown consumer behavior. Additionally, from the results in [29], consumer behavior classification is not valuable as it is not identifying different consumer behavior types, it is not robust, and it depends on social media interface structure.

The bit-series computation model is introduced by the authors in [30]. The ensemble model extracts the signatures utilizing a range of identification-based stochastics. Computed signatures are mostly seen in consumer behavior interaction structures. Therefore, false detection rates are reduced. The authors in [31] presented a basic regular expression-induced signature utilizing caterpillars. The presented model induced computed values and entailed several steps: recognizing the highest projecting series utilizing orientation methods, removing noisy portions, and creating regular expression.

#### 2.4.2. Behavior-Based Consumer Behavior Classification Models

Behavior-based classification models monitor the behaviors in the interaction network. Based on the monitored behaviors, the reviewing consumer is determined to be actual. This model has three portions: mining behaviors, creating features, and classification through machine learning models [32]. When extracting behaviors, interaction data, shared messages, and posts are utilized. Behaviors are extracted by computing the order of the message sharing and their frequency. Activities are categorized, series are computed, and features can be attained. Granting the interaction structure fluctuates over time, and its overall behavior will not have changed entirely. Hence, numerous consumer behavior types are identified by employing the suggested model. Additionally, consumer behavior has remained formerly unknown, an is predicted.

Consumer behavior classification employing the graph method is clarified by the authors in [33]. Consumers are converted into a graph such that each vertex denotes a consumer, and the edge denotes a transition among the posts. Using the result of a consumer wording as an input, the links among social media posts are computed. The graph was mined and associated with the existing graph models. The graphs were defined as consumer or non-consumer behavior. Additionally, new actions that are detected during the social media analysis, were dynamically defined in the graph. The authors in [34] presented a centric model in which consumer behavior interactions are unlike from non-consumer behaviors. By employing the interaction variances, behavior structures were generated from the graph nodes. Further, by employing these sequences, consumer and non-consumer behavior classes were produced.

#### 2.4.3. Stochastic-Based Consumer Behavior Prediction Model

Stochastic classification is a composite classification model that utilizes diverse models together. This classification model uses prior knowledge of rules and machine learning models [24]. Stochastic models employ both sentiment and behavior features to produce rules. Founded on the generated rules, consumer signatures are formed and utilized to regulate diverse consumer behavior as well as formerly unseen consumer behavior. The model learns by employing definite parameters for validation and irregularities. Even though the hit percentage in identifying unknown consumer behavior is in elevation.

The authors in [25] presented a deep learning consumer behavior classification model. The model can detect formerly unseen consumer behavior variants. The authors in [26] clarified a stochastic signature method. In this model, parameters are produced of particles that can be consumer posts, comments, shares, and reviews.

#### 2.4.4. Model Checking Consumer Behavior Prediction Model

In model checking classification, malicious parameters are extracted by employing nonlinear temporal models to detect the parameter dependencies, which are defined as disclaimers [32]. Consumer behavior parameters are mined by employing the association rules of different actions that utilize unseen behavior. To mark the input as reviewing consumer behavior, the features that are extracted are compared with the former provisions. This presented model is resilient to secrecy and can identify new portion of the consumer behavior variants.

The authors in [34] presented a confirmation model to identify malicious consumer behavior. In the ensemble model, malicious activities were defined by employing a predicate logic specification model from the assembled social media posts. If the method controller properly identified the specification, the investigated case is defined as consumer or non-consumer behavior. In this model, consumer behaviors are identified among friends of the same consumer types. Additionally, new, unknown but comparable consumer behavior variants are identified. Agreeing to the method, a checking algorithm detected consumer behavior semantic features more precisely than usual classification models, and this enhanced the precision of the classification. A positive consumer behavior classification model that employed a checking algorithm was introduced by the authors in [31]. The ensemble model can classify various consumers. The model mined features from posts and comments and routinely authenticated them employing the former defined specifications. They employed a novel specification model, NSM. The research depicted good results; the model could classify several types of consumers.

A technique is proposed in [30] to identify consumer behavior. This model declined the checking difficulty to regulate a Büki pushdown model with figurative factors. Consumer behaviors are transferred into pushdown stacks, and then, by employing the stack predicate logic, the consumer behavior activities are detected. At the final stage, the consumer behavior is mined by comparing it with the pushdown specifications. The presented model is resilient to stealth faulty identification and detects consumer behavior with high precision.

#### 2.4.5. Deep Learning Consumer Behavior Detection Model

Deep learning models are extensions of machine learning that are trained from samples and take over from convolutional neural networks. Deep learning models are employed in representation processing, autonomous vehicles, speech recognition, and also consumer behavior classification. The deep learning classification method performs well with high accuracy and decreases the parameter dimension, but it is not resilient to hacking [12]. Moreover, forming hidden layers requires more time; constructing more hidden layers will enhance the accuracy slightly while consuming more time. Deep learning models are not yet widely employed in consumer behavior classification, and thus, more research is required to precisely validate this model. The deep learning consumer behavior classification methodologies that are found in the literature are described below.

A deep learning consumer behavior classification model employing two-dimensional consumer behavior features is presented in [30]. The presented model has three central phases: In the first phase, five matching parameters are mined from reviewing consumers and non-consumer cases; in the next phase, three deep and dropout layers are constructed; in the last phase, the outputs are computed by employing the calibrator metric. At this phase, the approximation of whether the social post is consumer behavior or not is classified. From their results, the model performs well, with 96.7% accuracy with high sensitivity and F1-score.

The authors in [31] explicated a deep learning network they defined as a multitasking training model for consumer behavior identification. In the presented model, reviewing samples are trained using dynamic learning. Multitasking training permits the model to pre-train even at shallow layers, and the network utilized optimization techniques to reduce the counts of epochs and decrease errors; the researcher claimed that the model accomplished good accuracy in comparison with other deep models. However, the accuracy of the network cannot have increased by having additional deep layers. The authors in [32] introduced a mixed deep learning model to identify zero-period consumer behavior. The presented model employed multiple hidden layers using a Boltzmann network and short-term memory. It has two stages: model learning and parameter tuning. In the learning phase, training is performed by extracting features using a supervised fashion. At this phase, the features of each post are extracted. Then, in the parameter tuning stage, labelling is performed to split consumer from non-consumer behavior.

Deep learning classification models are operative when identifying consumer behaviors that can be misled by normal posting with harsh words, which yields miss-identification. For example, argumentative posts can deliver misleading inputs to and generate wrong consumer classification. Additionally, true consumers can be unidentified by shifting some characters in the wording. In our ensemble consumer behavior method, essential constraints are employed to diminish the impacts of such reviews.

## 3. Material and Methods

**Figure 1:**
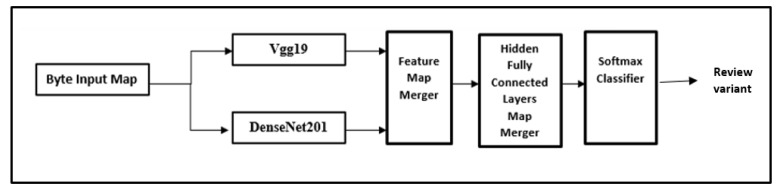
The ensemble consumer behavior classification model.

In this paper, we are presenting our ensemble consumer behavior learning model. This platform is an ensemble deep-CNN model. The ensemble deep-CNN platform as depicted has several stages (Figure 1). In the initial stage, the consumer behavior data are gathered by exhaustive database mining. In the second stage, unimportant and important consumer behavior parameters are extracted employing transfer learning CNN. The final stage starts a supervised learning module.

The following subsections describe consumer behavior representation and the deep learning model. In the consumer behavior representation, we will present the ensemble consumer behavior variants in binary. In the model description section, the ensemble consumer behavior classification model is explained.

### 3.1. Consumer Behavior Representation as a Binary Map

Various methods are introduced to transform binary file into maps [29,30,31]. This research utilizes the representation of the consumer behavior binary maps [27]. The required goal is to represent binary maps as a binary representation. Based on our algorithm, the consumer behavior binary file is represented as 8-bits vectors of unsigned integers. Then the binary number B is transformed into its number value employing Equation (1). At the end, the value is combined into a 2D array M and construed as a binary representation. The dimension of matrix M depends upon the consumer behavior file size

(1) $$B={ь}_{7}+{ь}_{6}+{ь}_{5}+{ь}_{4}+{ь}_{3}+{ь}_{2}+{ь}_{1}+{ь}_{0}\;\overset{\;\;\;\;}{\to }\;B\;={ь}_{7}\times 2^{7}+{ь}_{6}\times 2^{6}+{ь}_{5}\times 2^{5}+{ь}_{4}\times 2^{4}+{ь}_{3}\times 2^{3}+{ь}_{2}\times 2^{2}+{ь}_{1}\times 2^{1}+{ь}_{0}\times 2^{0}\;$$

### 3.2. The Ensemble Model for Consumer Behavior Prediction

The ensemble method defines a platform for consumer behavior prediction. This platform is an deep CNN structure. Our model of the ensemble platform is previously depicted in Figure 1, with consecutive stages: assembly of consumer behavior data, deep CNN structure, training, and testing phases. A flow diagram is depicted where the pre-trained CNN represents a feature extractor module. The first five layers exhibit FC layers for the training module and the Softmax layer.

Primarily, consumer behavior data are composed from several databases such as Facemg [27], BIG-D15 [28], and TwitD [29]. The consumer behavior databases are detailed in the following subsection. Then, the ensemble deep CNN model is depicted. There is a pre-processing phase: the procedure of predicting a proper deep learning architecture to embed in it the consumer behavior classifier. It is revealed in pre-processing that the ensemble technique can deliver better precision. An ensemble module that encompasses both DenseNet201 and Vgg19 CNNs is constructed employing transfer learning networks.

The DenseNet201 [32] network is a CNN with 50 layers with 5 convolutional layers. The DenseNet201 CNN has Maxpooling functions, classifier and FC neural layers. The DenseNet201 network has 25 million hyper parameters as described in Table 1. Vgg19 [33] is a prominent CNN network for large-scale representation recognition. Vgg19 has an architecture of several deep layers; the primary layers are convolutional neural layers. Vgg19 has two normalization operations, two pooling layers, and a final classifier, as depicted in Table 1. The description of DenseNet201 is depicted in Table 2.

Transfer learning has been examined for facing the various challenges faced in the classification model such as computational time cost and large data dimension. Transfer learning perform feature extraction process employing pre-trained CNN. Then, the classification procedure is performed with support vector machine (SVM) or with a Softmax classifier. This process is accustomed for the ensemble Deep CNN to aid with the above challenges.

The ensemble model associated CNNs with an identical weight to generate a representation map. The learning is then accomplished to attain high precision. The steps are as follows: The transfer learning procedure is completed with the DenseNet201 and Vgg19 networks using three databases in the training phase [31]. In the second phase, the parameters extracted by the DenseNet201 and Vgg19 networks are joined to produce the feature vector. This produced vector has 2048 dimensions. The features produced by the pre-trained DenseNet201 and Vgg19 are extracted from the final FC layer and depicted in T 6 and 7. Then, the joined feature vector is delivered to the Softmax and the FC layers to achieve normalization. Afterwards, the Softmax classifier produces seven outputs that consist of categories of consumer behaviors, and the FC layers encompass 2048 nodes. The last layer targets the enhancement of the learning ability of the ensemble deep-CNN. Finally, the ensemble deep learning model is tested by employing comprehensive databases in the training module. The description of the ensemble model is depicted in Table 3.

## 4. Results

### 4.1. The Implementation Process

**Figure 2:**
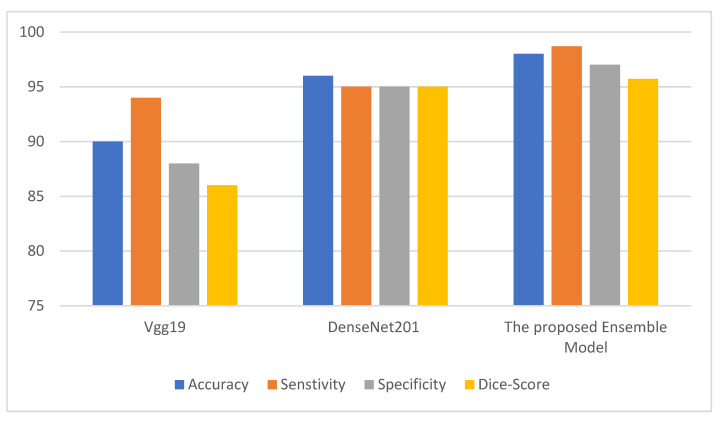
The performance of Vgg19, DenseNet201, and the ensemble models for the Facemg database.

**Figure 3:**
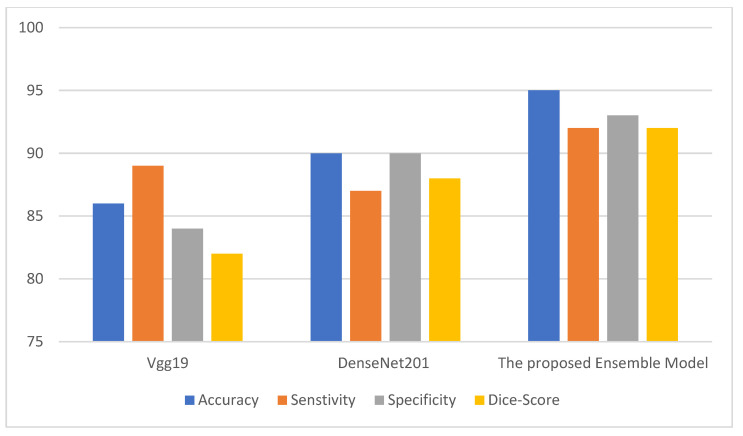
The performance of Vgg19, DenseNet201, and the ensemble models for the BIG-D15 database.

**Figure 4:**
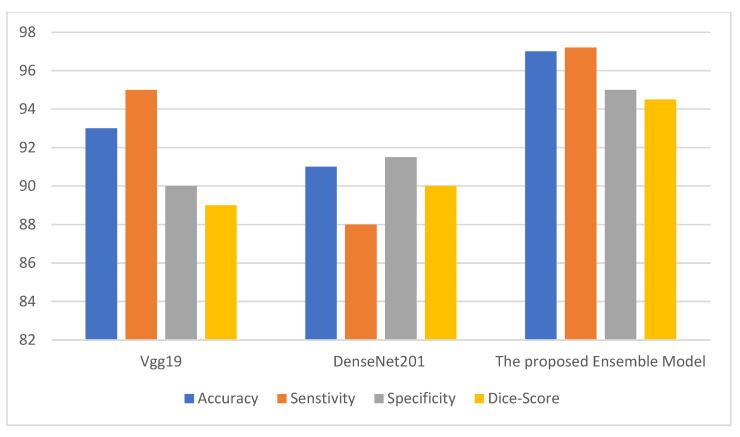
The performance of Vgg19, DenseNet201, and the ensemble models for the TwitD database.

This section describes the implementation process, the experiments, and the evaluation of the ensemble deep-CNN model. The experiments are performed on Intel Core i19 running at 9.6 GHz with 64 GB RAM. Python language was used to implement the model. Data are partitioned into training and validation databases randomly: 70% of the data for training, 15% for validation stage and 15% for the testing stage. The training process of the deep-CNN model was completed in 29 h for 80 epochs on average. Metrics such as accuracy, sensitivity, specificity, and F-score are used. These metrics are calculated as follows:

(2) $$\mathrm{ACC}=\frac{\mathrm{TP}+\mathrm{TN}}{\mathrm{TP}+\mathrm{FP}+\mathrm{FN}+\mathrm{TN}}$$

(3) $$\mathrm{SEN}=\frac{\mathrm{TP}}{\mathrm{TP}+\mathrm{FN}}$$

(4) $$\mathrm{SPEC}=\frac{\mathrm{TN}}{\mathrm{FP}+\mathrm{TN}}$$

(5) $$D=\frac{2*\mathrm{TP}}{2*\mathrm{TP}+\mathrm{FP}+\mathrm{FN}}$$

where TP denotes the count of true positives, FP denotes the count of false positives, TN denotes the count of true negatives, and FN denotes the count of false negatives. The metrics above are used to infer the performance of the ensemble model. The ensemble model is compared with two deep neural models. Figure 2, Figure 3 and Figure 4 depict the metric evaluation of the introduced model using an ensemble of Vgg19 and DenseNet201 deep models for each database. Table 1 depicts the initial parameters values of the selected configuration for the ensemble deep architecture used for the Facemg, BIG-D15, and TwitD databases as depicted in Table 4.

### 4.2. Benchmark Database

Experiments are performed on three benchmark databases. These are Facemg, BIG-D15, and TwitD. These databases are described below.

The Facemg database [27] has 9000 consumer behavior instances. Each single consumer behavior instance in the database fits into one of 20 consumer behavior classes. The count of instances fitting into a consumer behavior class varies across the database. The consumer behavior classes include different types of weapons (13 classes), bombs (5 classes including asking how to make a bomb or purchase materials related to bombs), suicide reviews and killing wordings (2 words).

The BIG-D15 database [28] contains 22,000 consumer behavior instances belonging to 9 classes include different types of weapons (7 classes), bombs (one class), and suicide reviews (one class). Similar to the Facemg database, the count of consumer behavior instances over defined groups is not equally spread. A single consumer behavior instance is mapped to one eight-bit map representing a hexadecimal number representing the class number. We used the bytes to form a consumer behavior representation in our simulation.

The TwitD database [29] has 9000 consumer behavior instances for training and 5000 consumer behavior instances for testing fitting into to 20 consumer behavior classes. Each class has 450 instances for training and variable instances for testing. The consumer behavior classes are the same as the classes of the first database.

### 4.3. Evaluation

Assessment metrics depict the performance of the ensemble consumer behavior classification technique. The direct outcome of the classification model is a score to comprehend the accuracy of a model [18]. Accordingly, different performance scores stated in the results are employed to depict the efficiency of the presented models. The performance scores are accuracy and the Dice metric. Figure 2, Figure 3 and Figure 4 depict the performance of the Vgg19 and DenseNet201 deep models and ensemble models for the Facemg, BIG-D15, and TwitD databases. In agreement with these charts, it can be specified that the ensemble model performs other deep learning architectures. Our model performance also depicts comparable results for the three databases, while the results for the compared two deep learning models fluctuate considerably for the three databases. The aforementioned settings indicate that our model is more reliable and has higher accuracy in comparison with the other models.

Additionally, consumer behavior variants are examined using the metrics. Table 5, Table 6 and Table 7 depict the confusion matrices for the BIG-D15 database for nine consumer behavior classes of Vgg19, DenseNet201, and the ensemble models.

Performance rates for each consumer behavior class are verified with the statistics. It is detected that our ensemble model gives higher results for all consumer behavior classifications excluding casual wording class. The DenseNet201 model delivers a higher classification of the consumer behavior variant compared with other models.

We also compared the ensemble model with the state-of-the-art models. Table 5, Table 6 and Table 7 depict the accuracy results for the Facemg, BIG-D15, and TwitD databases for the ensemble model and other models, respectively. It should be clarified that the ensemble model performs better than the state-of- the-art models.

### 4.4. Discussion

In this research, we presented a novel deep learning model to predict different consumer behavior trends using an ensemble architecture This research was focused on the integration of two pre-trained optimized learning algorithms. This model maintains four phases of data gathering, deep modeling, training, and model evaluation. The ensemble model is tested on three social media databases. The experimental results proved the efficiently of consumer behavior trends classification with high precision that outperforms recent models in the literature.

#### Implications

This study contributes to the literature on consumer behavior trends classification in a number of ways. Firstly, we present an ensemble deep learning model of the consumer behavior trends such as recurrent purchases and loyalty in replying to the social media marketing content. The main contributions of our model are: defining unique parameters through data mining techniques from the consumer behavior data for the specified classes. Additionally, the parameter dimensionality is reduced considerably for faster learning and classification time.

This research also contributes to enterprises who reflect using social media as a marketing channel. The experimental results recommend to digital marketing personal the significance of using social media to influence consumer behavioral trends. Based on the first set of experiments, testing depicted that our scoring model of consumer behavior variants has an important impact on defining consumer satisfaction through social media content sharing. For future research based on our findings, we can encourage and design an interaction marketing model and apply deep learning model on consumer interaction in a more efficient way.

The second set of experiments, which computed the true positive and true negative rates as well as testing kappa coefficient, proved the precision of our model compared with the ground truth and that it attains higher sensitivity and specificity than other deep learning models.

Designing digital marketing strategies based on our scoring technique (from the extracted parameters), based on the deep learning and mining approaches, the quality of enterprises towards consumers can certainly be developed.

## 5. Conclusions

Consumer behavior classification models effectively identify consumer behavior variants that represent serious consumer behavior in the social media domain that can represent real-life consumers. Unknown people behind the screen with different languages and wordings make the consumer behavior classification a difficult process. Our research ensemble a new merged learning model that efficiently identify consumer behavior classes. The ensemble model employs comprehensive pre-trained models that depend on the transfer learning model. The data on consumer behavior groups were collected by employing several exhaustive databases. Then, the features are mined, and the parameters are computed by employing transfer learning models. Additionally, the ensemble model achieves deep parameter extraction.

The central role of our proposed hybrid model is to unite two optimized deep learning models. The ensemble model is tested and validated on Facemg, BIG-D15, and TwitD databases. The suggested ensemble model is compared versus the joined models individually. The experiment results established that the ensemble model can efficiently predict consumer behavior with high accuracy and Dice score. It is also found that our model is effective and decreases the feature representation space. The ensemble model was compared against other deep learning models. The experiments attained revealed the improvement and reliability of our model over other models. On the other hand, a few consumer behavior instances were not predicted properly. This is because those consumer behavior variants employed unseen mystification wording and depicted the same features with several consumer behavior variants.

## Figures and Tables

**Table 1:** The description of Vgg19 network [33].

Layer Type	Properties
Input Layer	512 × 512 representations
Two Convolutional	64 × 3× 3
Maxpool	Max pooling
Four Convolutional Layer	128 × 3 × 3
Maxpool	Max pooling
Four Convolutional Layer	256 × 3 × 3
Maxpool	Max pooling
Four Convolutional Layer	512 × 3 × 3
Maxpool	Max pooling
Four Convolutional Layers	256 × 3 × 3
Maxpool	Max pooling
Three Fully Connected Layers	4096
Softmax	Softmax
	Output consumer class

**Table 2:** The description of DenseNet201.

Layer Type	Properties
Input Layer	512 × 512 feature representation map
Two Convolutional Layers	112 × 7× 3
Average pooling	Average
Dense block 56 × 56	6× 3 × 3
Average pooling	Average
Dense block 28 × 28	12 × 3 × 3
Transition layer 28 × 28	1 × 3 × 3 + Maxpooling
Dense block 14 × 14	12 × 3 × 3
Transition layer 14 × 14	1 × 2 × 2 + Maxpooling
Dense block 7 × 7	48 × 3 × 3
Softmax	Softmax
	Output consumer class

**Table 3:** The description of the ensemble model.

Block 1	Block 2
Input Layer	Input Layer
Two Convolutional Layers	Two Convolutional
Maxpool	Average pooling
Four Convolutional Layers	Dense block 56 × 56
Maxpool	Average pooling
Four Convolutional Layers	Dense block 28 × 28
Maxpool	Transition layer 28 × 28
Four Convolutional Layers	Dense block 14 × 14
Maxpool	Transition layer 14 × 14
Four Convolutional Layers	Dense block 7 × 7
Maxpool	
Three Fully Connected Layers	
Softmax	Softmax
Feature Map Merger	
Fully Connected Layers	
Softmax classifier	
Output layer	Consumer Varient

**Table 4:** Parameters of the Facemg, BIG-D15, and TwitD databases.

Database Parameters	Facemg	BIG-D15	TwitD
Batch size	64	32	32
Dropout rate	0.4	0.4	0.4
Epoch count	80	70	80
Learning rate	0.006	0.006	0.006
loss function	Cross entropy	Cross entropy	Cross entropy

**Table 5:** Statistics metrics for the compared models for the Facemg dataset.

	Vgg19	DenseNet201	Our Ensemble Model Features Merging
TP + TN	0.91	0.915	0.98
FP + FN	0.09	0.085	0.02
Kappa coefficient (inter-qualitative reliability)	0.309	0.411	0.514
Mean square error	0.412	0.413	0.211

**Table 6:** Statistics metrics for the compared models for the BIG-D15 dataset.

	Vgg19	DenseNet201	Our Ensemble Model Features Merging
TP + TN	0.91	0.915	0.98
FP + FN	0.09	0.085	0.02
Kappa coefficient (inter-qualitative reliability)	0.309	0.411	0.514
Mean square error	0.412	0.413	0.211

**Table 7:** Statistics metrics for the compared models for the TwitD dataset.

	Vgg19	DenseNet201	Our Ensemble Model Features Merging
TP + TN	0.91	0.915	0.98
FP + FN	0.09	0.085	0.02
Kappa coefficient (inter-qualitative reliability)	0.309	0.411	0.514
Mean square error	0.412	0.413	0.211

## Data Availability

The data presented in this study are available on request from the corresponding author.
